# The Works of John Hunter, F.R.S.; with Notes

**Published:** 1837-04

**Authors:** 


ART. XI.—*The Works of* JOHN HUNTER, F.R.S ; *with Notes.* Edited by JAMES F. PALMER, Senior Surgeon to the St. George's and St. James's Dispensary, &c. *In four Volumes, illustrated by a Volume of Plates in Quarto.* Vol. I.—*London*, 1835. 8vo. pp. 643.

THIS volume has been published so recently, (although bearing, we know not why, the date of 1835, both on its title-page and its preface,) that there is left neither time enough of our trimestral period, nor space enough of our allotted pages, to do it that kind of justice which so important a work has a right to claim. Our readers must have found out, ere now, that we think articles in Reviews may have other merits beside the rapidity with which they follow the publication of the works on which they are founded; and, least of all, are we likely to fall into so open a snare in the case of such a work as that now before us. The name of John Hunter must demand from medical writers, in all succeeding times, the truest homage; and the publication of the first edition of his collected works is an event calculated to arrest the attention of every one who is capable of appreciating true excellence in science. Although we shall, therefore, take more ample time to review the personal character, the discoveries, and general merits of this great man, we cannot allow the present opportunity to pass without recommending this edition of his works to the earnest attention of our readers. It is a book which ought to be in the library of every medical man. We consider the profession under great obligations to Mr. Palmer for having at length removed from the literature of this country the reproach of having no collection of the writings of an author who was unquestionably, to use the words of Mr. Lawrence, "the greatest man whom this country has produced in medical science, without excepting even the immortal discoverer of the circulation; perhaps the greatest man, in the combined characters of physiologist and surgeon, that the whole annals of medicine can furnish."

The present volume contains the Life of the Author, compiled from original sources, and embracing an account of the Hunterian Museum, by Drewry Ottley; and Surgical Lectures, delivered in the year 1786

and 1787, and collated with several manuscript copies, with Notes, by the Editor. The subsequent volumes are announced to follow speedily, and their contents will be arranged as follows:—Vol. II. Treatise on the Natural History and Diseases of the Teeth, with Notes, by Thomas Bell, F.R.S. F.L.S. F.G.S., Lecturer on the Teeth and on Comparative Anatomy at Guy's Hospital. Treatise on the Venereal Disease, with Notes, by G. G. Babington, Surgeon to St. George's Hospital, and formerly Surgeon to the Lock Hospital.—Vol. III. Treatise on the Blood, Inflammation, and Gun-shot Wounds. Miscellaneous Practical Papers, collected from different Transactions, with Notes, by the Editor.—Vol. IV. Observations on certain Parts of the Animal Œconomy. Croonian Lectures on Muscular Motion, delivered before the Royal Society in the years 1776—1782. Miscellaneous Papers on Physiology, Zoology, and Comparative Anatomy, extracted from the Philosophical Transactions and other Works, with Notes, by Richard Owen, F.R.S. F.L.S., and Hunterian Professor to the Royal College of Surgeons in London.

